# Situating anticipation in everyday life: Using sensory methods to explore public expectations of synthetic biology

**DOI:** 10.1177/0963662518808694

**Published:** 2018-10-25

**Authors:** Robert Meckin, Andrew Balmer

**Affiliations:** The University of Manchester, UK

**Keywords:** anticipation, creative methods, futures, public engagement, public participation, public understanding, sensory methods, synthetic biology

## Abstract

Public involvement in technological anticipation is a common feature of contemporary sociotechnical innovation. However, most engagements abstract sociotechnical futures, rather than situating them in the everyday practices in which people are routinely engaged. Recent developments in synthetic biology have established the potential for ‘drop in’ replacements for ingredients in consumer products, particularly in flavour and fragrance markets. This article explains how a sensory methodology can be used to explore citizens’ everyday experiences and how these can be used to ground anticipation of possible sociotechnical futures. The article uses a socio-historical approach to analyse and compare two practice domains – caring for families and hygiene and personal care – to show how biosynthetic futures can disrupt existing relations between people, objects and ideas. The implications for conceptualising publics in synthetic biology and for approaches to public engagement and participation are discussed more broadly.

## 1. Introduction

Public involvement in *ex ante* technology assessment is a common feature of contemporary science and technology innovation and figures prominently in the governance of emerging technologies. There exists a rich international history of scholarship that has experimented with ways to elicit public opinion about technological futures and feed findings into development processes (Barben et al., 2008; Burgess and Chilvers, 2006; Karinen and Guston, 2010; Schot and Rip, 1997). However, while locating knowledge in everyday environments is a tenet of Science and Technology Studies (STS) research (Marres, 2015; Michael, 2006; Wynne, 1996), it has been under-developed in work on anticipatory public engagement. Furthermore, sensory phenomena have been especially neglected. To contribute to these debates, we explore the potential for engaging the sensorium, particularly the olfactory and tactile, as a way to understand how people already make sense of particular molecules from within existing, mundane practices, and how this might inform people’s responses to possible applications of biotechnology.

Synthetic biology is an interdisciplinary area of biotechnological research in which long-standing and recently developed genetic engineering tools and techniques, alongside engineering and design principles, are employed in the hope of ‘making biology easier to engineer’ (Silver, 2009). Proponents claim synthetic biology has the capacity to speed up production of knowledge, transform manufacturing of foods, drugs and energy and boost economies (e.g. Osborne, 2012; Synthetic Biology Leadership Council, 2016). In 2015, the United Kingdom’s research councils funded six synthetic biology research centres (SBRCs), each with a different focus, to further research and industrial potential of the field. One of the SBRCs identified fine and speciality chemicals as its focus and researchers have successfully synthesised the popular terpenoid ingredient menthol by engineering an *Escherichia coli* metabolic pathway for its production (Toogood et al., 2015).

Menthol is conventionally derived from agricultural purification or chemical synthesis and is a widely used ‘minty’ flavour, which often produces a cooling sensation on contact with skin. Menthol, on its own and as a component of peppermint oil, is found in many consumer goods including toothpastes, mouthwashes, muscle rubs, balms, shower gels, shampoos, cold remedies, medicines, confectionery and chewing gums. Potentially, its production through genetic engineering means that *E. coli* and other microorganisms could be used in future to manufacture a wide range of flavours and fragrances for inclusion in myriad consumer products. Responses to biosynthetic menthol could, therefore, be an important indicator of publics’ sense-making in the broader context of consumer products derived from industrial biotechnology, especially in fragrance, flavour and related markets.

In the next section, we outline the public participation and engagement work that has been conducted in synthetic biology. We then describe our methodology, which situates anticipation of technoscientific innovations in people’s everyday practices and we describe five areas of menthol practices and select two to explore in more detail, showing how the possibility of biosynthetic menthol disturbs relations among the sociotechnical components of such practices. We conclude by explaining how this method addresses critiques of participatory anticipation work, elevating the ‘order of the game’ (Irwin et al., 2013), and by discussing the implications for public participation and engagement, arguing that further attention needs to be paid to the ways in which futures are anticipated from within everyday life.

## 2. Publics, engagement and synthetic biology

There are now well-documented problems in public anticipatory engagement practices. Many practitioners, for example, employ events such as consensus conferences, stakeholder workshops and citizen juries, which often use promissory narratives to elicit talk, and typically involve particular constructions and framings of futures (Adam and Groves, 2007). Indeed, experts often take the central role in framing issues and debates meaning that everyday ways of understanding science and technology are marginalised (Bogner, 2012). Attempts have been made to overcome such framing biases (e.g. Chilvers, 2007) but even here there is a problem regarding how we should determine what will count as relevant futures to consider (Krzywoszynska et al., 2018). Furthermore, such events are often held in research and educational spaces, divorcing publics from the everyday situations in which science and technology become salient (Gehrke, 2014; Marres, 2015). These issues are interrelated, since choices about where to hold participatory activities can influence the kinds of people who attend, the specific definitions of sociotechnical fields adopted, the possible applications considered, and thus the kinds of futures that are anticipated. In other words, it is now clear that the ways in which publics are conventionally convened through participation and engagement practices do not fit well with how people actually conduct their own decision-making with regard to the present and to the future, from within the routines of their everyday lives.

Many participants in the field of synthetic biology have been active in engaging publics. A recent workshop report noted the numerous ways synthetic biology communities interact with publics (Pallett, 2017). However, it is not clear how many of these activities are about anticipation and technology assessment, as opposed to education, entertainment or evangelism. That being said, two major studies are indicative of the broader trend in (and problems with) anticipatory engagement. In 2010, the Biotechnology and Biological Sciences Research Council (BBSRC) funded a public ‘Synthetic Biology Dialogue’, which remains the most widely publicised engagement event in the United Kingdom. The Dialogue used a series of workshops to elicit citizens’ responses, finding that participants were most concerned about the scientists’ practices and motivations (Bhattachary et al., 2010). The report included five questions that scientists should think through prior to and in the course of their research^1^ but these were applied to synthetic biology as a whole, meaning they had very little specificity and were difficult to connect to any particular, possible sociotechnical future in which the likely products of synthetic biology might figure. Similarly, a more recent survey of ‘the US public’ found that people do not feel informed about synthetic biology, have low factual knowledge, are equally divided as to whether synthetic biology presents risks or benefits, and tended not to use politics in their judgements (Akin et al., 2017). These findings are in keeping with the kinds of results often derived from such abstracted, general engagements.

Problematically, because the concept of synthetic biology is itself broad and contested, discussions of general futures can be even more unfocussed than in other fields, and large-scale surveys and expert consultations struggle to address the consequent variability in the field’s possible applications and anticipated outcomes. This means researchers have engaged with publics, but with a focus on measuring general ‘public perceptions’ or ‘awareness’ of a ‘fuzzy’ object, namely ‘synthetic biology’ in general (Pardo Avellaneda and Hagen, 2016). Relatedly, researchers and practitioners (in the United Kingdom) construct imaginaries of publics hostile to synthetic biology, again as an abstract totality, because of the perceived ‘failure’ of genetic modification (Marris, 2015). The fuzziness of how the field and associated futures are rendered within these anticipatory practices, combined with a desire to avoid public rejection of the entire enterprise, means that anticipatory work can often be guided towards demonstrating a performative support for the promises of synthetic biology writ large, rather than towards specific, more likely possible futures in which people have a particular and determinable stake.

More recent synthetic biology engagement work has, however, demonstrated more promise. The use of video narrations as discussion stimuli in museums, for example, may develop participants’ awareness of different positions on synthetic biology and underlying assumptions affecting attitudes in ways which could in turn, generate reflexivity of participants’ own positions leading to more nuanced deliberation about the ethics and futures of synthetic biology (van der Meij et al., 2017). This suggests using novel methods and resources may be a way to enhance public involvement in anticipatory technology assessment. In this article, we report on the use of such a methodology and explore a form of socio-historical analysis that builds an anticipatory strategy, overcoming the limitations articulated in these critiques. We show how such an approach can be used to understand people’s anticipation of the distributed, mobile and personal consumer goods that might result from technoscientific innovations in a rich, situated fashion.

## 3. Sensory engagement

**Figure 1.**
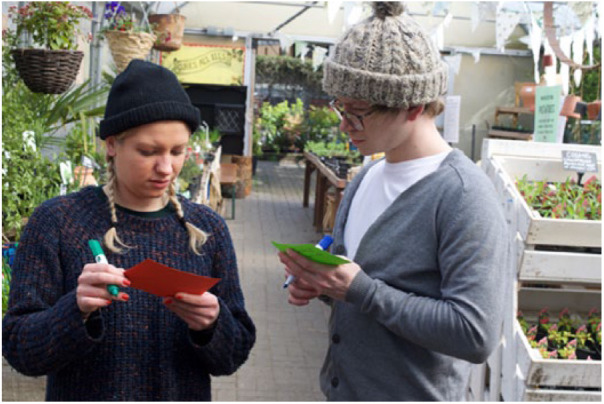
Two participants filling out postcards at the garden centre pop up.

**Figure 2.**
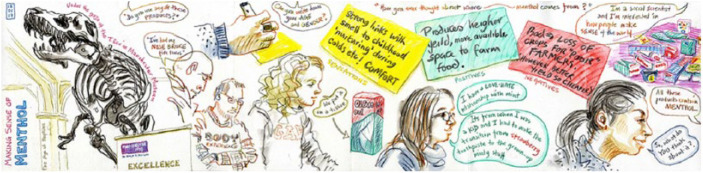
Artist’s concertina sketch of data collection during a museum pop up (Copyright held by Lynne Chapman).

In this section, we discuss how we elicited participants’ associations and memories of menthol sensations to explore relevant practices in an anticipatory way. Due to the contemporary distributions of menthol molecules, we were able to use existing menthol-containing products to elicit rich, descriptive and future-orientated exchanges with participants in different contexts. We experimented with different approaches to sensory engagement. We conducted five of what we called ‘pop up stalls’, which involved displaying menthol products on a table in public spaces and inviting people to try them, discuss their responses with us, and fill out coloured postcards depending on their responses. We held two stalls in university spaces and one each in a museum, a garden centre and a large shopping centre, collecting about 450 postcards (see Figures 1 and 2). We also used object-elicitation in two focus groups, seven interviews and nine ‘home tours’ where, as part of a home-based interview, participants showed us around their homes to menthol storage and use sites, such as a kitchens, bathrooms and bedrooms, showing us how they used these products as part of everyday routines.

The sensory methods we used emphasised participants’ bodies as sense-making apparatuses, foregrounding sensations, experiences and aesthetics to produce ‘somatic accounts’ (Vannini et al., 2012). People were able to talk without prompt in frank, sometimes comical or romantic ways, about intimate details and personal routines, which showed us how menthol figured in their existing, everyday experiences. We argue that our approach facilitates considerations of the future by situating them within the material and sensory phenomena routinely encountered in the present (Coleman, 2017; Tutton, 2017). We located anticipation in the spaces in which children’s skin, freshening the mouth, or invigorating the scalp are salient, which offered us a way to simultaneously off-set expert framings of futures, deflate fear and hype surrounding risks and benefits, and bring out the modes through which menthol is already meaningful, risky or beneficial in people’s mundane experiences. Our emphasis on bodily and affective responses locates understanding of innovation ‘in-the-moment’ of material interaction, rather than in the abstracted context of deliberative forums.

To collect data as people touched, tasted and inhaled the menthol products, we made audio recordings, took photographs, video and field notes and worked with an artist to produce ‘concertina’ sketchbooks (see Figure 2). The majority of our data were collected in autumn 2016 and spring 2017. Home tour and interview participants ranged from 5 years old (supervised by parents) to 60 years old. At the pop up stalls, however, it is likely that some people were older than 60, meaning we engaged with a diverse audience by combining a range of methods. The audio recordings of these encounters were transcribed and we integrated these with the field notes, diaries, photographs, videos and sketches in NVivo to thematically code them. We discussed the codes and possible connections with our wider research group (of eight people). Then, as part of the writing process, we made new connections and checked ideas that we developed with each other and against the transcripts, notes and visual data. Through this process, we identified five domains of practices in which menthol figures, which we outline below.

## 4. Mentholated practices

Menthol-containing consumer products sit on supermarket shelves in medicine, personal care and confectionery aisles, and on impulse buy displays near points of sale. The products span, on one dimension, from not-quite-foods to not-quite-pharmaceuticals. Theories of practice have shown how a range of elements are involved in the creation and performance of practices, including the materials used, the skills and know-how required, and the morals and aims of the practices (e.g. Reckwitz, 2002; Schatzki, 2002). We adapted Hand and Shove’s (2007) tabulated analysis of using a home freezer, and present the important components of five domains of practice in Table 1 below. The domains involved different formulations of menthol-containing products, spaces, skills, aims and morals.

**Table 1.** Practices including menthol-containing products.

Practice	Discourses or aims	Materials	Know-how or skills
Caring for families	Being a good parent (not using drugs or artificial ingredients), helping family members especially children to alleviate symptoms and get to sleep	Vapour rub, menthol oils or crystals.	Temporal and pragmatic aspects (evenings; not at school; only have to wash pyjamas) Assessing methods (e.g. hot bowls, tissues and rubs) Storage – out of children’s reach
Hygiene and personal care	Preparing self: arousing, sleeping and interacting with others. Being clean, fresh and not smelly Managing hunger or eating healthily	Toothpaste, mouthwash, shower gel, shampoo, mints and chewing gum. Chewing gums	Anticipating timings (e.g. before or after foods) Physical skills of lathering, rinsing and so on Accessibility, for example, have in car to stave off hunger so travellers do not snack on long drive
Modulating bodily functionalities	Alleviating symptoms Also used to restore or increase concentration or focus Restoring muscle function	Sweets, inhalers, tissues and rubs Chewing gums and mints Muscle rubs	Use with or instead of pharmaceuticals Discretion in professional or social situations
Pleasure	Treat self when socialising	Menthol cigarettes. Mint leaves, mint liqueur and drinks	Smoking and drinking alcohol Cocktail-making
As a focus of sociality	Interacting and bonding	Vapour rub and balms	Used as challenges and jokes, especially in performances of masculinity

Participants were often surprised at the range of products in which menthol was an ingredient. This surprise points to both the ubiquity and invisibility of menthol – it is so commonplace it is inconspicuous, which resonates with Wynne’s (1991) observation that ‘science’ almost disappears in everyday life and with more recent work on ‘ordinary’ material assemblages (Miller and Woodward, 2012). Even when people engage with science in an explicit way, such as in hackerspaces, engagements are not necessarily about radical critique or politically revolutionary; they can be more mundane, like filling leisure time or being part of a community (Davies, 2018). Similarly, the practices we describe do not tend to be politically radical, explicitly transformational or critical of their social milieu. Rather, the use of menthol in family care, hygiene, functionality, leisure and sociality practices enacts ritualised understandings of the relations between people, family members, social obligations and professional lives. There is, therefore, an everyday ethics and politics embedded in the routine use of menthol goods. In the next section, we explain how explorations of mundane material practices can be a site for people to make sense of biotechnological futures and show how the everyday connects to broader socio-political structures shaping anticipation of science and technology. We chose two of the more dominant domains in our data for deeper discussion – (1) caring for children and (2) hygiene and personal care.

## 5. Caring for children

When participants saw and smelled Vick’s brand vapour rub, and other similar products, they often began accounts with ‘mmmm, I love that’, or, ‘that reminds me of my grandparents’, or, ‘that takes me back to being ill when I was smaller’. Following these immediate emotional comments, people talked about the way that parents and grandparents used muscle rubs when the participants were ill, particularly if they were ‘snotty’. At bedtime, carers would rub ointment onto children’s chests and backs with the aim of helping them breathe more easily through the night. We found a similar phenomenon with Olbas Oil and related inhalants. Inhalants often involve adding the oil to hot water and either setting in a bowl to allow the mixture to diffuse through a room or, for a more intense effect for adults, bending over the bowl with a towel over one’s head. Through object elicitation, we can see that the smell and sensation of menthol are intimately connected to ways that people recollect their childhoods, and particularly with the way care is enacted and recalled. Many parents described using menthol on their children’s bodies, too, and talked about how practices of care using menthol are passed on through families, suggesting that menthol figures within ‘inheritable’ family schemes of tacit and practical knowledge.

**Figure 3.**
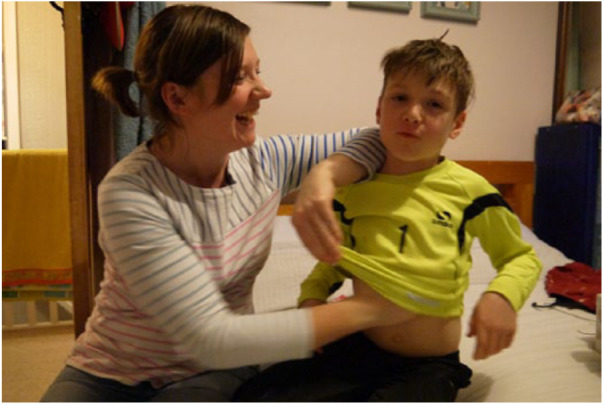
Jen and her son demonstrating how they use a vapour rub.

Participants connected menthol-containing products to memories and moralities highlighting how sensations of chemicals can be deeply intertwined with people’s biographies and the way they make sense of current moral commitments, such as their approach to parenthood. For example, using menthol to care for children was important in creating physical connections between parent and child, as demonstrated by Jen during Home Tour 3 (see Figure 3).

The ways parents tended to use vapour rub was consistent: when their children were ill or ‘sniffly’ they would rub the ointment onto their chests and backs. Furthermore, menthol has become tied up with the routine of the Western day, and how illness is managed to preserve that routine: it figures as an evening thing; it is about settling-down and preparing for sleep; it is linked to work through the protection of parents’ energies for the next day; and a key part of the caring schedule, because the evening and settling for bed is often when explicit rituals of care and demonstrating affection are conducted (e.g. reading a story, putting on the pyjamas, cuddling and singing). When asked about using menthol, Jen, pictured above, said,

… this to me feels almost like an alternative therapy, something alternative to giving them [my children] paracetamol so for me this is much [pause] I would be quite happy to lather them in this because I would feel it is not going to do you any harm whatever benefit you get, whether I put it on your feet or your chest or your back or whatever, if it is going to help you, whereas I wouldn’t want to do that with paracetamol … and I think I would feel a bit more maybe I shouldn’t do that if it is an artificially created product because I wouldn’t trust it as much … this [vapour rub] feels like it is natural to me … but it probably isn’t natural, but that is just my perception of it … Drug free … because you are not putting things [pause] like I said I don’t think it gets into your blood stream, so you are not ingesting it. (Jen, 33 Home Tour 3)

In the quotation above, Jen explains that she considers vapour rubs to be desirable for use on her children because they are safer and do not carry the same possibilities of side-effects as do pharmaceuticals like paracetamol. Her account is reflexive and nuanced, however, because even as she ties trust to a notion of what is ‘natural’ she acknowledges that this may not be factually accurate. ‘Nature’ then, is being deployed as a proxy for the understanding that the vapour rubs are not entering (her children’s) bodies. The category of natural, here, is thus a resource Jen uses strategically in her practice of parenthood. The bodies of her children are managed through routines of protection from harm and with respect to the potential of harm such that milder, more established remedies are preferred over unknown or uncertain elements that can be categorised as artificial and potentially harmful.

Indeed, Vick’s Vaporub is marketed as a family company, specifically tying it into family histories of tacit, practical knowledge of care. Originally called Vick’s Family Remedies, the brand went under the slogan ‘The only thing more powerful than a mother’s touch’ after it was bought by Proctor & Gamble in 1985. Currently, Vick’s Vaporub is marketed as ‘safe cough relief so the family can sleep’ (Vicks, 2018), implicitly invoking the contemporary connection to work routines. Thus, Proctor & Gamble trade on the familial, safe and tactile elements of menthol’s effects as part of vapour rubs. Perturbing the arrangements of production, particularly if regulators introduced clear synthetic biology labelling or new testing regimes, may mean that such a company would need to consider carefully the uptake of the new method, corporate branding, and its ability to play-on and capitalise on socio-material family histories.

We can also consider this issue, briefly, from a socio-historical perspective. ‘The child’ became an object of protection from cruelty at the turn of the twentieth century in Victorian England. This transformation included the establishment of legal frameworks and the National Society for the Prevention of Cruelty to Children (NSPCC), the idea of which had been imported from New York City, in 1889 (Ferguson, 2004). These formal developments were accompanied by changing vocabularies. Subsequent legislative and cultural changes have meant that the notion of ‘child protection’ has a continually shifting conceptual history including associations with maltreatment, neglect, cruelty, abuse and so on. The definition of child abuse in the United states, for example, changed between the 1960s and 1970s from physical attack or injury to causing some deficit, which would affect the development opportunities of a child (Hacking, 1991). This is significant for our case study, since what it meant to be a good parent within the West also changed as the concepts of abuse and maltreatment expanded to include failures of positive interventions, such as not providing optimal conditions for growth, emphasising parents responsibilities to be active in demonstrating effective childcare. This means that the moralities surrounding parenting practices are dynamic and changing, but have been on a trajectory of increasing the protection of children’s bodies and emphasising the active management of providing optimal care and opportunities to flourish within family practices.

Returning to our research data, parents can be seen as enacting these more recent concepts by trying to navigate a way through children’s illnesses on one hand, and ethics of intervention on the other. Some parents described using menthol instead of, or as a first choice in care, rather than using pharmaceuticals. Vapour rubs and inhalants were thus ‘mild’ interventions that represented an attempt to ameliorate a child’s symptoms, which in turn may enable them to sleep and be better prepared for school the next day. It also meant avoiding the use of more active compounds that parents attached to possible side effects, unless more direct medical intervention became necessary. From this understanding, when Jen described using menthol because it is a ‘natural’ remedy, what may be most significant is not her specific (and perhaps scientifically inaccurate) definition of ‘natural’, but the implications of her deployment of the term. In anticipating biosynthetic menthol, Jen constructs her children’s bodies as zones for protection and demonstrates her moral responsibility as a mother to optimise their experience of health, sleep and school. It follows that the potential of biosynthetic menthol disturbs these relations because it brings novel, uncertain elements into the frame which she feels ill equipped to negotiate and which trouble her performance of the role of active and attentive mother. Strategic distinctions between natural and artificial are not, therefore, evidence of poor understanding which can be corrected but, by bringing existing moralities attached to these categories to bear on the anticipation of synthetic biology futures, a skilful navigation of this socio-political terrain.

In the context of caring for children, then, we can anticipate several issues with biosynthetic menthol. The first is that because children’s bodies are objects of protection, and families are sites of safety, there needs to be a good reason to change from a conventional configuration of objects deemed as safe and natural to a novel configuration, and that reason has to make sense within the set of norms and values embedded within these practices. The second issue follows the first: promises regarding sustainability and economic benefit do not appear to be as relevant as concerns about safety when it comes to children or demonstrations of good parenting. Indeed, the established connections between the oil and pharmaceutical industries (Vaporub’s main ingredient is petroleum jelly) are unexamined or hidden. Finally, scientific knowledge producers are not considered experts of relevance to this problem, for science is not where most parents turn to inform themselves of what is regarded as parenting best practice and scientific, economic and environmental norms and values are not immediately attached to these caring routines. Instead, parents turn to their families, their wider parenting networks and their health practitioners to inform their practices of care, connecting instead to the ethics and values associated with family life. In the next section, we outline and highlight contrasts between what we described here and what we found in hygiene and personal care practices.

## 6. Hygiene and personal care

Menthol is situated in everyday life in various rituals of personal cleaning and hygiene. These activities involve brushing, swilling, wringing, wiping, sponging, gargling and so on, and participants typically, though not always, performed them on themselves, and our participants did not tend to connect them to other family members. Where participants in the previous section spoke of using vapour rubs at night to soothe and relax children, participants using menthol as part of skin and scalp cleaning, such as in shower gels or shampoos, often found products too invigorating for use at night. In this context, people described using menthol in the morning to wake up and become more alert. This follows a recognised trend where aromatics are often used to affect one’s wakefulness and emotional state in specific ways (Classen et al., 1994; Shove, 2003: 104). Participants at our pop up stalls recorded the relations between cleaning, menthol and affect, for example,

Colgate – clean teeth. Feels nice (no details, Garden Centre)

Makes me feel more refreshed and cleaner. (Female, 12, University ‘taster’ day)

The smell makes me feel fresh. (Female, 16, Dept. Open day)

While these postcard comments are brief, they show that menthol and minty flavours are an important part of feeling clean. Odourising and deodorising the body in particular ways has long been a crucial component of rituals of cleansing, though what particular smells signify, including their intensities, continually shift. Mint, in ancient Rome, was thought to revive the spirit and ameliorate stomach aches (Classen et al., 1994: 41). However, in eighteenth century France, aromatics began to be an object of suspicion where the ‘use of powerful perfume cast doubt upon a person’s cleanliness’ and more subtle odours became fashionable (Corbin, 1986: 68). Furthermore, where bathing and cleaning were social affairs taking place in public baths, showering and bathing have become increasingly individualised, conducted in personal bathrooms, which have co-located previously separate practices of excretion and ablution. As science and medicine’s understandings of infection developed, cleaning has also become about removing invisible microbes and preventing disease transmission. Although the organisation of cleaning has changed significantly, ideas of relations between smells and diseases still inform contemporary cleaning practices alongside the ideas of germs (Shove, 2003). In our study and in contemporary Western culture more broadly, cooling peppermint and menthol smells are associated with freshness, cleanliness and hygiene.

In our longer exchanges, participants tended to be more expansive and detailed in their responses. In the quotation below, Dani, 33, who was of Chinese heritage, spoke about how minty sensations were connected to oral hygiene:

… the market is overly saturated with mint. You have no choice, really… I have learned to appreciate that minty taste in the morning … Funnily enough my friend, as a joke, got me a cupcake flavour toothpaste that I’m hesitant to use, and, I think in some ways, because toothpaste is saturated with mint, you associate that nice minty taste with being clean and anything else is sort of counterintuitive to that feeling, so you don’t feel like it’s [your mouth’s] as clean. (Dani, 33, object-elicitation interview 3)

Dani comments on the oral hygiene market, saying there is a limited choice in terms of product flavours but that she has learned that the sensations of using menthol, its cooling effect and minty taste, make her mouth feel clean. Dani is dubious about using the ‘joke’ flavour toothpaste because the affective reason for toothbrushing – feeling clean – will not be achieved. What Dani describes is an embodied understanding of what it means to be clean, where bodily sensations are intimately connected to the affective dimensions of hygiene practices, the cupcake flavour toothpaste is funny precisely because it is out of place. Thus, there are relations between bodily sensations, emotions, materials and discourses in the domain of personal care with which the possibility of biosynthetic menthol can intersect, potentially complementing but also disrupting hygiene practices.

Indeed, in our data, participants made sense of the possibility of biomanufacturing using everyday experiences of hygiene. Participants in the pop up stalls deployed their knowledge of infections and germs, for instance:

It’s a good idea to make menthol this way as it’s more sustainable. I’m not put off by the fact it comes from bacteria. (Female, 17, Dept. Open day)

Bacteria is bad (disease). (Male, 13, University ‘taster’ day)

In the long-term would the bacteria affect any part of your body? (Male, 40, Museum)

It could, as the first quotation shows, not result in a rejection of biosynthesis, but in an evaluation of different concerns and claims of potential, where broad discourses of conserving resources and environments could be prioritised over one’s own ethical or affective concerns. This is different to the caring practices above, where children’s wellbeing came before other economic or environmental concerns.

Moreover, the notions of ‘good’ and ‘bad’ bacteria are fairly simplistic, and possibly originate in advertising campaigns, such as those for the Yakult brand of probiotic drinks, as well as the contents of the previous national curriculum (Department for Education and Skills (DES) and Qualifications and Curriculum Authority (QCA), 2004). In contrast, for example, Kim, 26, a sufferer of several conditions including chronic fatigue syndrome and nausea, and frequent user of mint and alternative therapies, explained how she imagined other people would respond to biosynthetic menthol. In the quotation below, she constructs futures where consumers have the options for choosing between two similar products, one produced agriculturally and one produced biosynthetically, each with clear labelling:

I am Vegan, and I have had many discussions with other people about where their stuff comes from, and people say they care where their stuff is from, but they don’t. For example – if you had this shower gel and this one [holding up two bottles] – here right and you said, ‘this has got 1,000 real – oh my god it says it on it! – this has got all these real mint leaves on it and this one says, ‘grown in a petri dish from *E. coli*’ – nobody would buy that [the bacterial one], and everyone would buy this [the mint leaves one]. Even if this was 50p or a pound cheaper, or even if this was free, people would be like, ‘oh, I am not putting *E. coli* on my body. I can remember the Cucumber gate, like, it’s [putting biosynthetic menthol on me is] not happening’. But really, you know, if you said to somebody, do you know that this [agriculturally derived] one has been tested on a rat and it’s got like fifty-two different chemicals in it, and this one has just one like it’s just from *E. coli* – people don’t actually really care what is in it. (Kim, 26, home tour 7)

**Figure 4.**
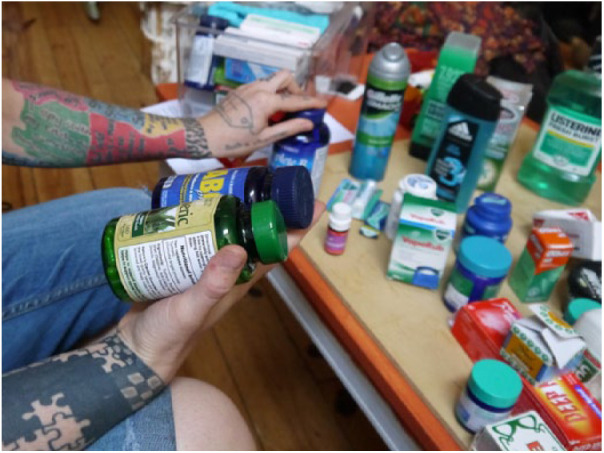
Participant holding different products as they compare labels and brands.

Kim explains how people’s attitudes to products can be affected by branding and labelling, showing a critical assessment of the market, like Dani above, but which was not prominent in the previous section (see Figure 4). Kim imagines people who would not apply *E. coli-*derived menthol to their body because there is an established and trusted option derived from mint plants. On the other hand, she imagines another scenario where people might find out the cocktail of ingredients in a plant-derived product has been tested on animals. Thus, when she says that ‘people don’t care what’s in it’, she implies that the chemicals in and of themselves are not problematic, but that the processes of production, testing, labelling and advertising matter because they make different connections to people’s morals, desires, feelings and experiences. A situated understanding of menthol such as this is in tension with a chemical understanding of menthol whereby molecules are identical no matter their context or provenance. In scientific communities, this is often coupled to the assumption that if only people understood their sameness they would accept production by other (e.g. biosynthetic) means.

What we have seen is that practices can be a useful unit of analysis to explore the possibilities of synthetic biology in an anticipatory manner. Comparing caring for families and hygiene practices, it is possible to highlight different relations with practices. The ways that bodies are important, the particular morals and aims surrounding each practice and the skills and knowledges available to participants were different. The possibility of biosynthetic menthol, understood in these contexts, potentially altered the relations between the different elements in various ways. What is important for our article’s contribution to the field of public understanding of science is that contextualising people’s anticipation in everyday practices led to explorations of futures that were framed by the values of mundane life, rather than the socio-political implications developed by experts and brought to bear in anticipatory forums, surveys and dialogues. In the next section, we summarise our findings and briefly discuss implications for public participation and imaginaries of publics in synthetic biology.

## 7. Concluding discussion

We have supplemented public participation in technology assessment by demonstrating how a sensory methodology can situate discussions of technological futures in the embodied present. We described how we elicited people’s responses to mentholated products and detailed domains of practices in which those products were used. We discussed two of these areas of practice to show how a social scientific analysis can help elucidate how participants engaged with the possibility of biosynthetic menthol. Our analysis may well have specific relevance to other flavours and fragrances, and perhaps biosynthetic consumer products more generally.

The approach we have described offers several strengths over some more common engagement and anticipation strategies and builds on recent, more creative efforts. First, it contributes to existing research diversity by showing how it is possible to enrich deliberative and anticipatory methods through a sensory, situated engagement with possible futures. What we observed was that using object elicitation as a stimulus was a quick way to establish dialogue with participants, whether they were passers-by at pop up stalls or being interviewed in their living room. In this way, we helped invoke the forms of expertise that people are more likely to employ when dealing with science and technology in their everyday lives, rather than those they use in more traditional deliberative contexts. Second, we tackled the issue of ‘fuzziness’ in technoscience futures by focusing our research on the specific molecule ‘menthol’ and its associated markets, its possible futures and the everyday practices in which it is already involved. Third, we have shown that comparing analyses of domains of practices (see the Table 1 on p.11) can help elucidate subtle differences in configurations that make particular resources available for people to use to inform their anticipation. Fourth, the ways publics make sense of anticipated futures will be clearly different when everyday practices, values and meanings are used as the source of publics’ perspectives. Indeed, our participants sometimes found it hard to explain in words the specific sensations (e.g. ‘I can feel the smell in the back of my nose’) and often quickly developed somatic responses into moral, political or reflexive accounts, concurring with findings elsewhere that the senses are best understood as embodied, entangled with one another, and with the socio-political, and as connections between materials and abstractions (Mason and Davies, 2009).

Starting from an understanding that a chemical is entangled with affect, ethics and sensations may have important implications for what constitutes a ‘sensible’ or ‘responsible’ reaction to biotechnological innovations. In this regard, we add our research to slowly developing efforts towards more contextualised engagement with science in the ‘real world’ (Delgado et al., 2011; Gehrke, 2014; Wiek et al., 2013). We also suggest that such an effort opens the door to a range of creative methodologies already used in sociology and other disciplines to engage with the mundane factors, which inform people’s incorporation of science and technology into their everyday experience. There is potential that the methods and findings we have described can alter innovation processes in a more coherent fashion. However, our approach places sociological work in a privileged position for the interpretation of public anticipation of technology. This has implications for the subsequent responsibility of representing such interpretations to scientific communities and decision makers (Barben et al., 2008; Williams, 2006). How to conduct such policy and governance work through the use of deep qualitative engagement with situated publics remains open to experimentation.

With regards to publics, there are two key points. The first is that we conceptualised publics as organised around sets of practices, rather than as specific groups, to show how menthol is implicated in social life across many dimensions, including biographies, families and bodies. Thus, a chemical understanding constructs menthol molecules as identical structures whereas an analysis of practice suggests that menthol acquires different meanings and significance through its use in different practices, for example, it can be about protection, freshness or cleanliness. Second, publics are often constructed in narrow political ways, convened for the purposes of ‘public dialogue’. Instead, we have discussed how publics are already connected to a range of powerful practices, social structures and embodied socio-temporal routines, including things like company identities, branding products, the Western history of child protection, family norms and values, everyday routines of work and sleep, and so forth. This indicates that we need to imagine and explore the future in a more nuanced fashion, and – especially when engaging publics in such deliberations – to do so in ways which are empirically grounded in lived experience rather than abstracted from the day-to-day. It is not to say that there is a problem with engagement with publics which explicitly seeks to help bring about a future in which synthetic biology plays a role, *per se*, but that this should be done in ways which are informed by a deeper appreciation of publics’ practices, not simply their ‘attitudes’ regarding risk and benefit.

## Notes

1. Synthetic Biology Dialogue questions: (1) What is the purpose? (2) Why do you want to do it? (3) What are you going to gain from it? (4) What else is it going to do? and (5) How do you know you are right?
